# Diffuse T‐Wave Inversion After Chest Pain: A Catecholamine‐Mediated Takotsubo‐Equivalent

**DOI:** 10.1111/anec.70114

**Published:** 2025-10-14

**Authors:** Zhong‐Qun Zhan

**Affiliations:** ^1^ Department of Cardiology Shenzhen Guangming District People's Hospital Shenzhen P. R. China

Li et al. (Li et al. 2025) report a 38‐year‐old woman who presented 20 days after the onset of chest pain with diffuse, deep T‐wave inversion (TWI) and QTc prolongation in leads I, II, III, aVF, and V_2_–V_6_, but in whom the acute‐phase ECG had not been captured. Cardiovascular magnetic resonance (CMR) showed no late gadolinium enhancement. Both TWI and QTc normalized within 3 weeks of tumor resection. I would like to place this observation in the patho‐electrocardiographic framework of Takotsubo cardiomyopathy (TTC).

## 1 Diffuse TWI as a “Footprint” of Undocumented STE

In TTC, the ECG evolves through three rapidly successive stages: (i) hyper‐acute ST‐segment elevation with upright T waves, (ii) Q‐wave formation while STE persists, and (iii) widespread TWI with QT prolongation that resolves as oedema disappears (Zhan, Wang, Sclarovsky, et al. 2013; Zhan, Wang, Nikus, and Sclarovsky 2013). CMR demonstrates that the territory of acute transmural oedema colocalizes exactly with the leads that initially show STE; the same anatomical area exhibits TWI a few days later, and both changes vanish together on follow‐up (Eitel et al. 2011). Although Li's patient was first recorded at Day 20, the lead‐by‐lead distribution of TWI (I, II, III, aVF, V_2_–V_6_) perfectly matches the classical STE map of TTC, and the absence of late gadolinium enhancement confirms reversible injury rather than necrosis (Li et al. 2025; Eitel et al. 2011). These features strongly imply an undocumented STE phase mediated by paraganglioma‐driven catecholamine surges.

## 2 Normal Wall Motion Does Not Exclude a TTC‐Equivalent Mechanism

The authors excluded TTC because echocardiography and CMR performed late in the course showed no regional wall‐motion abnormality. However, the classic “apical balloon” is only one phenotype. Focal mid‐ventricular, basal, or segmental variants are well described and may retract within 5–7 days (Medina de Chazal et al. 2018). The 20‐day time window in Li's case exceeds the typical oedema life span. Thus, the ventricle had already recovered, yielding a false‐negative mechanical image. The preserved electrical signature (TWI) while contractility has normalized is precisely what is observed in subclinical or recovered TTC.

## 3 Imaging Timing Is Critical

CMR or echocardiography beyond Day 7 may miss wall‐motion abnormalities even when electrical markers persist. Future CCM case series should report the exact interval between symptom onset and imaging. This will likely reveal that many “functionally normal” hearts actually traversed a transient TTC‐like state.

In conclusion, Li's case exemplifies a catecholamine‐mediated reversible TTC‐equivalent. The electrical footprint (diffuse TWI) is preserved, while the mechanical abnormality has already resolved. Recognizing this sequence prompts earlier plasma/urinary metanephrine screening in patients with unexplained, diffuse TWI and avoids the pitfall of discarding TTC solely based on late, normal imaging.

## Conflicts of Interest

The author declares no conflicts of interest.

## Data Availability

The data that support the findings of this study are openly available in figshare at https://doi.org/10.1111/anec.70114.
